# A Scoping Review of Physiological and Behavioural Pain Responses of Ultramarathon Runners

**DOI:** 10.3390/sports14100427

**Published:** 2026-10-01

**Authors:** Marie Delalay, Sabina Hotz Boendermaker

**Affiliations:** Institute of Physiotherapy, School of Health Professions, University of Applied Sciences ZHAW, 8400 Winterthur, Switzerland; sabina.hotzboendermaker@zhaw.ch

**Keywords:** pain, ultramarathon, physiological pain responses, behavioural pain responses

## Abstract

Pain during ultramarathon races is almost inevitable. Effective pain responses are associated with enhanced race performance. This scoping review synthesises the existing literature on physiological and behavioural pain responses during ultramarathon races and explores the association of these responses with performance. Following the PRISMA Extension for Scoping Reviews (PRISMA-ScR), we included 31 studies. We organised the extracted data into five overarching categories: neurophysiological mechanisms, running mechanics, medication use, physical therapies, and performance outcomes. Our results show that some ultramarathon runners exhibit exercise-induced analgesia, whereas others exhibit inflammation-driven pain sensitisation. Changes in running mechanics during races may be a response to present or anticipated pain. In addition, many ultramarathon runners take pain medication during the race, mainly to prevent or reduce pain. During ultramarathon races, massage interventions may relieve pain. Finally, ultramarathon runners’ performance may decline in the presence of pain. Among ultramarathon runners, a variety of pain responses are associated with race performance. Because effective pain responses may lead to better performance outcomes, future research should investigate the causal mechanisms linking pain experiences, pain responses, and race performance.

## 1. Introduction

Ultramarathons are running events that cover distances longer than the traditional marathon (42.195 km). Ultramarathon races are organised worldwide, with a marked concentration in Europe and the United States. In many countries, the number of events has risen over the last two decades, suggesting growing interest and participation in ultramarathon races [1].

Participation in these physically and mentally challenging events is driven by diverse motivations. Commonly reported motivations relate to conventional ideas of performance and success, such as achieving personal goals and pushing personal limits [2]. In addition, other reported motivations do not directly pertain to race performance: ultramarathon runners seek to stay healthy, lose weight, be in nature, strengthen their self-esteem, find meaning and purpose, and meet like-minded people [2]. Furthermore, ultramarathon participants, like recreational road runners, may be driven by affective and embodied experiences, such as freedom, escape, elation, and pleasure [3,4].

Motivations are closely linked to subjective race experience, including but not limited to race performance. Race performance generally deteriorates when runners are prevented from racing in accordance with their goals and expectations—owing, for instance, to fatigue, injuries, or pain [2,5,6,7,8]. While pain is central and inevitable in ultramarathon races, more effective physiological, behavioural, and mental pain responses are associated with better performance, whereas less effective pain responses are associated with poorer performance [2,4,5,6,7,9]. A recent scoping review suggests that mental pain responses encompass thought processes, psychological traits, pain expectations, and cultural discourses. However, causal relationships between mental pain responses and race performance have yet to be established [10].

Long-distance running can cause inflammation-induced pain that leads to pain sensitisation [11,12]. Pain sensitisation is characterised by increased responsiveness to a given nociceptive signal in peripheral and/or central pathways [13]. It may manifest as allodynia, pain from normally non-painful stimuli, or as hyperalgesia, an increased pain responsiveness to a normally painful stimulus. Related to hyperalgesia is a lowered pain threshold, identified as the minimum stimulus intensity at which a subject perceives pain [13]. In contrast, pain tolerance is defined as the maximum intensity or duration of a pain-producing stimulus that a subject is willing to accept, and is thus heavily influenced by psychological factors [13].

Pain sensitisation and amplification may also be associated with reduced functioning of descending pathways, as assessed using conditioned pain modulation. Conditioned pain modulation reflects the net effect of descending inhibitory and facilitatory pathways by comparing responses to a painful test stimulus before, during and after the application of a remote painful conditioning stimulus [14].

This scoping review aims to (1) synthesise the existing literature on physiological and behavioural pain responses of ultramarathon runners and (2) examine the association of these responses with race performance. These insights will expand knowledge in sports science and pain science. These insights may also inform strategies for responding to pain more effectively and, ultimately, enhance race performance.

## 2. Methods

We followed the PRISMA Extension for Scoping Reviews (PRISMA-ScR) [15]. We designed the review protocol iteratively. We resolved disagreements, mostly amicably, during face-to-face meetings.

Working independently, both authors screened English-language publications related to “ultramarathon” and “pain” from four online databases: PubMed, ScienceDirect, Scopus, and Web of Science (see Table 1 and the top part of Figure 1). Because titles and abstracts of scientific articles typically include all keywords of the body text, we refrained from full-text search. We included articles published between 1 January 1900 and 20 August 2026. During data charting, we iteratively conducted backward and forward citation searches. As such, these searches constituted the fifth data source. At the end of this stage, we identified 128 non-duplicate publications.

We filtered these 128 publications by applying four sequential inclusion criteria as shown in Figure 1. First, we excluded 9 publications unrelated to ultramarathons. For example, publications focusing on triathlons were excluded. Second, we excluded 33 publications that were not peer-reviewed original research articles, e.g., resulting in the exclusion of literature reviews and book chapters. Third, we excluded 45 articles in which neither results nor findings pertained to pain responses during the race. For instance, articles that described injuries and diseases during races without referring to pain were excluded. Fourth, we excluded 10 articles that did not substantially address physiological or behavioural pain responses. For example, we excluded articles that reported solely on mental pain responses. As a result, we included 31 articles on physiological and behavioural pain responses during ultramarathon races.

During data charting, we manually extracted in each study the objectives, research design, findings on physiological and behavioural pain responses, and number of study participants. If a study focused on a specific race, we also extracted the names, locations, distances, ascents, durations, and terrain types. Finally, we organised the data into five overarching categories: neurophysiological mechanisms, running mechanics, medication use, physical therapies, and performance outcomes.

## 3. Results

### 3.1. Study Characteristics

Table A1 displays the charted data. We identified 16 descriptive observational studies (i.e., case series or single-case reports), 9 analytical observational studies (i.e., cohort, cross-sectional, or case–control studies), 5 qualitative studies, and 1 experimental study. Studies have a mean sample size of 87 (±138) participants, with half of the studies including 24 participants or fewer. Six studies are not race-specific, while most race-specific studies examine races in Europe and North America. A variety of terrain types is represented, including mountain trails, desert paths, and loop running tracks. Race distances range from 44 to 330 km. Eleven studies focus primarily on pain, while the remaining twenty examine pain only in relation to their primary focus.

### 3.2. Neurophysiological Mechanisms

Five studies report on peripheral and central neurophysiological mechanisms in ultramarathon runners: one on hypoalgesia, two on pain threshold, and two on pain tolerance [11,12,16,17,18]. Based on our synthesis of the studies, ultramarathon runners may experience exercise-induced hypoalgesia and inflammation-driven pain sensitisation. Furthermore, higher pain tolerance levels are associated with greater ultramarathon running activity.

Ultramarathon runners may exhibit exercise-induced hypoalgesia. In Hoffman, Lee et al. [16], ultramarathon runners rank the intensity of pain induced by a pressure pain stimulator applied to the index finger at the same pressure before and immediately after a 161 km running race. Subjects are divided into two equal-sized groups based on finishing time: the faster runners and the slower runners. When pre- and post-race values are compared, there is a significant decline in pain intensity ratings among faster runners, but no significant change among slower runners. Hoffman, Lee et al. [16] hypothesise the following: (1) faster runners experience more exercise-induced hypoalgesia than slower runners; (2) this difference is caused by the exhaustion or insufficient activation of systems responsible for exercise-induced hypoalgesia in the slower runners; and (3) this exhaustion or insufficient activation is due to an exercise intensity that is too low.

In Agnew, Hammer et al. [11] and Agnew, Roy et al. [12], study participants’ pain threshold, which is measured by a pressure algometer, decreases from the start to the end of the race when measured without a conditioning stimulus. Interestingly, when the participants are divided into two age groups, this decrease is only observed in the younger runners [12]. Agnew, Roy et al. [12] hypothesise that the difference between younger and older runners is due to inflammatory responses, fitness levels, and psychological traits. In both studies, inhibitory pain modulation conditioned via cold water decreases from the start to the end of the race. In both studies, the authors infer that this decrease is a manifestation of central and/or peripheral pain sensitisation following an inflammatory response.

Higher pain tolerance levels are associated with greater ultramarathon running activity. In Roebuck, Urquhart et al. [18] and Freund, Weber et al. [17], ultramarathon runners have higher immersion times in the cold-pressor test than the control group. The authors infer that ultramarathon runners have a higher pain tolerance than the general population, and that higher pain tolerance is associated with greater ultramarathon running activity. They hypothesise that (1) ultramarathon running activity increases pain tolerance, (2) psychological and/or physiological mechanisms may explain this analgesic effect, and (3) underlying mechanisms include inhibitory conditioned pain modulation, exercise-induced analgesia, and analgesia from blood baroreceptor reflexes.

### 3.3. Running Mechanics

Nine studies examine the link between running mechanics and pain responses: seven on running pace and four on running style [4,6,7,19,20,21,22,23,24,25]. Based on our synthesis of the studies, ultramarathon runners modify both their running pace and style during the race, which may be a response to present or anticipated pain.

Ultramarathon runners monitor and adjust their pace, possibly to prevent or reduce pain. Participants in a 330 km running race ration their physical and mental resources to preserve a healthy mind–body state and prevent pain and injury [7]. They have diverse approaches to managing their physical and mental resources. For example, they modify their pace and take breaks. Likewise, ultramarathon runners in Holt, Lee et al. [20] monitor their pace to cope with stressors, such as pain. In Degache, Serain et al. [25], runners reduce their pace in the first part of a 330 km ultramarathon; the authors hypothesise that runners do so to prevent or reduce muscle damage and pain.

Additionally, ultramarathon runners in Antonini, Rochat et al. [6] and Schütz, Schmidt-Trucksäss et al. [24] prevent or relieve pain by reducing their pace. In Hanold [21], pace reduction is one of the responses to “bad pain”, which is defined as the pain that study participants interpret as a warning for potential or actual loss of function. Finally, in a 56 km running race, the odds of exercise-associated muscle cramping decrease with longer running time in the first half of the race after controlling for personal best times. This suggests that a relatively low pace in the first half of the race prevents pain caused by exercise-associated muscle cramping in the second half of the race [19].

Ultramarathon runners modify their running style during the race, which may be a response to present or anticipated pain. In Antonini, Rochat et al. [6], ultramarathon runners adjust their running style to relieve pain. For example, they put more weight on one leg, stick to trails, and avoid pebbles. Bennett, Ratzlaff et al. [4] report on a study participant consciously controlling her posture to alleviate acute pain. In Degache, Morin et al. [22] and Morin, Tomazin et al. [23], the running patterns of the study participants become safer and smoother as the race progresses. Aerial times, vertical downward displacement of the centre of mass, and peak vertical ground reaction force decline significantly from the start to the middle or end of the race. While emphasising that alterations of running patterns and spring-mass behaviours are mainly driven by structural muscle damage and neuromuscular fatigue, the authors in both studies also hypothesise that ultramarathon runners change their running patterns because they seek—consciously or not—either to reduce actual leg pain during phases of eccentric muscle activity or to prevent pain and injuries towards race completion.

### 3.4. Medication Use

Six studies report on the use of medication by ultramarathon runners in relation to pain: one on riboflavin and five on various medications [5,16,26,27,28,29]. Based on our synthesis of the studies, ultramarathon runners take pain medication during races, primarily for pain prevention and relief.

Riboflavin may reduce tissue damage and pain during an ultramarathon due to its protective effect on tissues. In a randomised controlled trial on the effect of riboflavin ingestion on pain during a 161 km running race, muscle pain intensity ratings of the riboflavin group during and immediately after the race are significantly lower than those of the placebo group [28]. It is known that prolonged physical exercise leads to oxidation; that oxidation causes tissue damage; that tissue damage typically causes pain; and that riboflavin can protect tissue from oxidative damage. Therefore, Hoffman, Valentino et al. [28] infer that pain is reduced because of riboflavin’s protective effect on tissues.

While studies agree that ultramarathon runners use pain medication during races, they report a wide variation in the proportion of runners who do so. Didier, Vauthier et al. [26] find that 28% of runners in a 160 km race and 11% in a 72 km race take medication during the race. The two major medication classes are non-steroidal anti-inflammatory drugs (NSAIDs; 9.8%) and other analgesics (6.7%). In Martínez, Aguiló et al. [27], 51.7% of finishers in a 112 km race, 39.8% in a 67 km race, and 17.7% in a 44 km race use NSAIDs during the race. In Hoffman and Fogard [5], at least 60.5% of finishers and at least 46.4% of non-finishers of a 161 km ultramarathon take NSAIDs or acetaminophen during the race. 86% of non-control subjects in Hoffman, Lee et al. [16] report taking acetaminophen or anti-inflammatory drugs during a 161 km running race. In Cole, Bontemps et al. [29], 53% of the study participants report NSAID use during training or competition. Estimates of the proportion of ultramarathon runners who take medication increase with race distance [26,27], probably due to increased muscle damage or pain sensitisation in longer races.

Interestingly, the main goals of taking pain medication are pain reduction and prevention. In Didier, Vauthier et al. [26], 29.6% of participants declare taking medication to reduce pain and 28.2% to prevent it. In Martínez, Aguiló et al. [27], 56.4% of participants use NSAIDs to prevent pain, 30.9% to relieve pain, 9.1% to treat injuries, and 3.6% to ease headaches. In Cole, Bontemps et al. [29], 74% of NSAID users report using them to “mask an existing injury” and 54% to “prevent performance deterioration.”

### 3.5. Physical Therapies

Two studies report on the impact of physical therapies on pain [30,31]. Based on our synthesis of the studies, massage interventions may relieve pain in ultramarathon runners during the race. In Visconti, Capra et al. [31], physical therapists provide massages to runners with delayed-onset muscle soreness during a 330 km mountain trail race. Pain intensity ratings on a 0–10 scale decrease significantly by 3.6 from pre- to post-massage. Similarly, in dos Santos Bunn, de Souza Terra et al. [30], participants in a 24 h race on a loop running track rate their pain intensity on a 0–10 scale before and after three different therapeutic treatments. Pain intensity ratings decrease significantly not only with massage (pre-treatment rating = 5.1 ± 2.2 and post-treatment rating = 1.9 ± 2.1) but also with stretching (pre-treatment rating = 4.5 ± 2.1 and post-treatment rating = 1.7 ± 2.0) and cryotherapy (pre-treatment rating = 5.0 ± 2.0 and post-treatment rating = 2.4 ± 2.3).

### 3.6. Performance Outcomes

Thirteen studies report on the association between pain responses and performance outcomes: eight on withdrawal odds and eight on finishing times and race rankings [4,5,9,21,29,32,33,34,35,36,37,38,39]. Based on our synthesis of the studies, pain is associated with performance deterioration, including race withdrawals, longer finishing times and poorer race rankings.

Race withdrawal is associated with pain. Various painful injuries and illnesses leading to race withdrawal are presented in single-case reports: acute cardiac problems causing chest pain in Sadowska-Krępa, Kłapcińska et al. [32], corneal freezing and abrasion causing ocular pain in Cope and Kropelnicki [33], oesophageal rupture causing chest pain in Pasternak, Ellero et al. [34], and painful injuries in Best, Barwick et al. [35]. In addition to acute pain, “bad pain”, which is defined as pain that warns of potential or actual loss of function, leads to race withdrawal for some ultramarathon runners [21,36]. In Alschuler, Krabak et al. [9], non-finishers report (1) higher worst pain than finishers and (2) larger changes in pain intensity and pain interference than finishers. Both observations suggest that pain represents a risk factor for ultramarathon withdrawal. Finally, in Hoffman and Fogard [5], the main reasons for race withdrawal are pain-related symptoms for 30.2% of the non-finishers: pre-existing injury or injury during the race (15.1%), blisters or “hot spots” on the feet (5.8%), muscle cramping (5.0%), and muscle pain (4.3%).

Besides withdrawal, performance deterioration such as longer finishing times and poorer race rankings is associated with pain experiences. Gajda, Samełko et al. [37] conjecture that knee pain in a participant in a 24 h running race leads to a lower ranking. Gatterer, Rauch et al. [39] infer that the performance of ultramarathon runners must be explained by a variety of factors, including pain. During the first half of a six-day ultramarathon in Bennett, Ratzlaff et al. [4], study participants seek to optimise traditional performance outcomes; during the second half, they prioritise meaning and purpose over performance.

In addition, pain interferes with race performance in Alschuler, Kratz et al. [38], Alschuler, Krabak et al. [9], and Rochat, Hauw et al. [36]. Hoffman and Fogard [5] find that many factors affecting the performance of both finishers and non-finishers relate to pain. These factors include blisters or “hot spots” on the feet, reported by 40.1% of finishers and 17.3% of non-finishers; muscle pain, reported by 36.5% of finishers and 20.1% of non-finishers; pre-existing or race-related injury, reported by 16.5% of finishers and 25.9% of non-finishers; and muscle cramping, reported by 11.4% of finishers and 15.8% of non-finishers. Finally, in Cole, Bontemps et al. [29], pain and discomfort from menstruation and menopause impair the performance of female ultramarathon runners.

## 4. Discussion

### 4.1. Perspectives from the Broader Literature

This scoping review synthesises 31 studies examining the multidimensional nature of pain during ultramarathon races and identifies complex interactions among neurophysiological mechanisms, running mechanics, pharmacological and therapeutic interventions, and performance outcomes. Evidence indicates that race participants may exhibit exercise-induced hypoalgesia and analgesia, adjust pace and gait in conjunction with pain, and use medication to manage pain. Pain and athletic performance are closely linked, as “bad pain” or insufficient coping strategies are associated with performance deterioration, such as race withdrawal.

Evidence suggests that exercise-induced hypoalgesia occurs with both high-intensity exercise and exercise performed at 60–75% of maximal oxygen uptake [40]. However, athletes and non-athletes differ in their pain perception. Generally, athletes have higher pain thresholds and higher pain tolerance levels and report lower pain intensity ratings than non-athletes [41]. Studies examining conditioned pain modulation with brain imaging reveal that central neural processing differs between athletes and non-athletes, suggesting greater endogenous central pain inhibition in athletes [41,42,43,44,45,46]. Moreover, athletes across different sports differ in their pain perception. For instance, endurance athletes exhibit greater inhibitory conditioned pain modulation than strength athletes, indicating greater endogenous central pain inhibition in endurance athletes [47]. Yet, many of these neurophysiological mechanisms in ultramarathon runners and athletes remain unexplained. Further research is needed to understand the relationships among pain perception, exercise, and endogenous pain inhibition.

Our synthesis of the included studies indicates that pain is associated with changes in running mechanics. Ultramarathon runners may respond to present or anticipated pain by adjusting their pace and style, a strategy supported by the teleological model of exercise regulation. Ulmer [48] demonstrates that athletes reduce muscle activation to optimise performance and manage discomfort, indicating that altering running style is an essential coping mechanism.

The interaction between pain, running mechanics, and neuromuscular impairment is complex. Musculoskeletal pain has been shown to disrupt neuromuscular control, triggering muscle imbalances and general fatigue that adversely affect running economy [49,50]. However, it is difficult to isolate the direct effects of pain from those of exercise-induced muscle damage on running mechanics. As increased running intensity or duration causes stiffness, swelling, loss of muscle function, and delayed-onset muscle soreness, the resulting mechanical alterations may occur concurrently with, rather than solely because of, the experience of pain. Therefore, future research must clarify the causal mechanisms linking muscle damage, nociception, and mechanical adaptation in the ultramarathon running context.

The literature’s estimates of the proportion of ultramarathon runners who take medication are not directly comparable. First, estimates for races held in Europe [26,27] are lower than those for races held in the United States [5,16]. This difference may be owing to different attitudes towards medication across regions. Second, only Hoffman and Fogard [5] report findings divided between finishers and non-finishers. Didier, Vauthier et al. [26] make no distinction between finishers and non-finishers, while Martínez, Aguiló et al. [27] and Hoffman, Lee et al. [16] consider only finishers. Third, studies examine different medication classes with distinct physiological effects. To make estimates comparable across future studies, methodologies will have to align, implying (1) the collection of relevant data, such as attitudes towards medication and (non-)finisher status, and (2) a consistent approach regarding the medication classes studied.

NSAID use is prevalent in elite and recreational athletes of many sports disciplines. Overall, NSAIDs can reduce acute pain, but do not improve performance [51]. When used inappropriately, NSAIDs have gastrointestinal, renal, and cardiovascular adverse effects, as well as potential interference with muscular adaptation. The prevalence of, and risks associated with, NSAIDs call for scrutiny of dosing regimens. In the future, challenges in health literacy within the athletic community should be addressed, and clinical and evidence-based guidelines should inform decision-making [52,53,54].

We hypothesise that the use of medication during ultramarathon races may be associated with pain intensity ratings and pain unpleasantness. Most studies included in this scoping review ignore pain unpleasantness. Because of the inherent subjectivity of pain, neglecting pain unpleasantness may oversimplify the complex nature of pain experiences, pain responses, and their causal associations with race performance. In a clinical context, pain unpleasantness ratings generally coincide with higher pain intensity ratings. By contrast, in an ultramarathon running context, in which pain is both feared and embraced, feelings of unpleasantness do not necessarily increase with higher pain intensity [4,41,55,56]. The study of pain unpleasantness in ultramarathon runners is scant and based on quantitative methods, such as rating scales and questionnaires [41]. As phenomenological research designs focus on conscious and lived experience of the participant without attempting to derive meaning or causation beyond the participant’s viewpoint, future research should rely on both quantitative and phenomenological approaches, including interviews and focus groups, to study pain unpleasantness in the ultramarathon running context and its association with pain experiences and medication use [57].

The possible pain-relieving effect of massage interventions on ultramarathon runners could be explained by at least two mechanisms. First, athletes’ positive expectations, formed by contextual cues and the patient–therapist relationship, enhance endogenous pain inhibition [58]. Second, massages activate low-threshold mechanoreceptors and non-nociceptive nerve fibres. According to the Gate Control Theory, the activation of these large-diameter fibres inhibits the transmission of nociceptive signals [59]. This mechanical stimulus may promote the release of endogenous opioids and modulate parasympathetic activity, thereby increasing the pain threshold and facilitating recovery.

In Hoffman and Fogard [5], finishers report higher absolute prevalence of blisters and muscle pain than non-finishers do, even though pain may lead to race withdrawal. We explain this apparent contradiction as follows. More intense pain-related symptoms such as blisters and muscle pain characterise finishers, while more intense pain experiences such as worst-pain ratings and pain interference characterise non-finishers.

The literature demonstrates that pain experiences may lead to performance deterioration. However, not only pain experiences but also fitness level, inflammation, neuromuscular fatigue, prior experience, training history, pacing strategies, and nutrition determine performance outcomes [39]. Conversely, performance outcomes may influence pain experiences; for example, a good ranking during a race boosts self-confidence, which results in endogenous pain inhibition [58]. As such, future research is needed to explore the causal mechanisms underlying performance and pain during ultramarathon races.

### 4.2. Limitations

This scoping review has three main limitations. First, some findings are corroborated by no or only one study. We found only one study on hypoalgesia [16], one on riboflavin [28], two on pain threshold [11,12], two on pain tolerance [17,18], and two on therapies [30,31]. Second, many studies have small sample sizes, which undermines their representativeness: half of the studies include 24 participants or fewer, and five are single-case reports.

Third, and most importantly, most study designs preclude both the establishment of causal mechanisms and the derivation of quantitative findings. As such, most of the findings are associations, and many cannot be granted statistical significance. This limitation also characterises the literature on mental pain responses of ultramarathon runners [10].

In addition, three limitations in the study of pain in ultramarathon runners are worth noting. The first limitation pertains to the use of rating scales to quantify pain intensity. Although scale-based pain intensity ratings have been thoroughly investigated and validated, they are subjective—as is pain itself—and subject to many biases: subjects’ language, sex, gender, and expectations (e.g., subjects expect researchers to administer painful stimuli below the damage threshold); main sport type (e.g., contact sport or endurance sport); and label of maximum pain (e.g., “worst pain imaginable” or “severe pain”) [41].

Second, the wide variety of pain locations (e.g., musculoskeletal, cutaneous, and visceral) and origins (e.g., injuries, fatigue, and illness) hinders comparisons across studies. Studies consider highly heterogeneous pain locations and origins, for example, eye pain from injury in Cope and Kropelnicki [33] and pain in the lower extremities from fatigue in Best, Barwick et al. [35], Degache, Morin et al. [22], and Morin, Tomazin et al. [23]. Third, studies diverge in how they distinguish and aggregate fatigue, injuries, and pain. Some study participants experiencing extreme fatigue or pace-reducing injuries refer to these experiences as pain. This points to a lack of a phenomenological exploration of pain in some studies [60,61].

## 5. Conclusions

This scoping review synthesises 31 studies on physiological and behavioural pain responses and their associations with race performance in the ultramarathon running context. We have organised these pain responses into five overarching categories: neurophysiological mechanisms, running mechanics, medication use, physical therapies, and performance outcomes.

We have found, first, that ultramarathon runners may experience exercise-induced hypoalgesia and inflammation-driven pain sensitisation, and that higher pain tolerance is associated with greater ultramarathon running activity. Second, ultramarathon runners adjust their running pace and style during the race, possibly in response to present or anticipated pain. Third, ultramarathon runners take pain medication during races, primarily for pain relief and prevention. Fourth, during races, massage interventions may relieve pain in ultramarathon runners. Fifth, pain may lead to performance deterioration such as race withdrawal, slower finishing times, and poorer race rankings. Among ultramarathon runners, pain responses—mental, physiological, and behavioural—are associated with race performance. Because effective pain responses may lead to better performance outcomes, future research should investigate the causal mechanisms linking pain experiences, pain responses, and race performance.

## Figures and Tables

**Figure 1:**
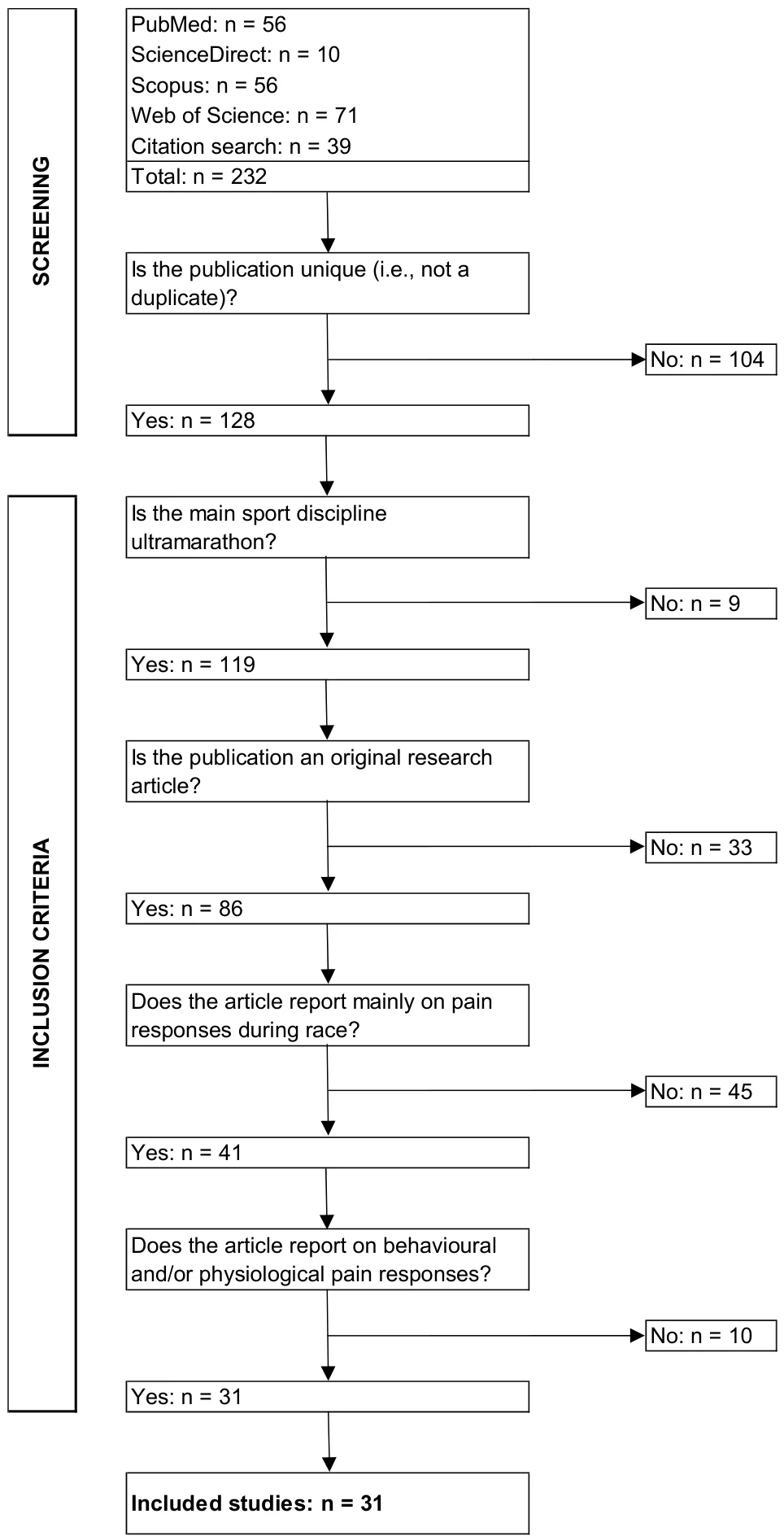
Workflow diagram used to select included studies.

**Table 1:** Databases, queries, and websites.

Data Source	Query	Website
PubMed	(ultramarathon[Title/Abstract]) AND (pain[Title/Abstract])	https://pubmed.ncbi.nlm.nih.gov/advanced/ (last accessed on 20 August 2026)
ScienceDirect	Title, abstract, keywords:ultramarathon pain	https://www.sciencedirect.com/search (last accessed on 20 August 2026)
Scopus	(TITLE-ABS-KEY (ultramarathon) AND TITLE-ABS-KEY (pain))	https://www.scopus.com/pages/home#basic (last accessed on 20 August 2026)
Web of Science	(TS = ultramarathon) AND (TS = pain), in all databases	https://www.webofscience.com/wos/alldb/advanced-search (last accessed on 20 August 2026)
Backward and forward citation search	Article title	NA

## Data Availability

No new data were created or analyzed in this study. Data sharing is not applicable to this article.

## Appendix A

**Table A1:** Charted data from included studies. Rows are ordered by research design, publication year and first author. Column “Study Participants” indicates the number of study participants.

Authors	Objectives	Race Description	Study Participants	Research Design
Hoffman, Valentino et al. [28]	To determine whether acute ingestion of riboflavin reduces muscle pain and soreness during and after an ultramarathon	Western States Endurance Run, California, USA; 161 km of mountain trails; 5500 m of ascent; Cut-off time of 30 h	32	Randomised controlled trial
Agnew, Roy et al. [12]	To investigate whether pressure pain threshold and conditioned pain modulation are different for older and younger ultramarathon runners	Wild Sebastian 100, Saint Sebastian 100 and Keys 100 ultramarathons, Florida, USA; 161 km of sandy trails, concrete sidewalks and gravel paths; Average finishing time of 26 h	37	Cohort study
Gatterer, Rauch et al. [39]	To investigate the association between laboratory-based performance parameters and performance times in a short (68 km) and a long (121 km) mountain ultramarathon	Südtirol Ultra Skyrace, Italy; 68 km (short race) and 121 km (long race) of mountain trails; 4260 m (short race) and 7554 m (long race) of ascent; Average running times of study participants: 12 h (short race) and 28 h (long race)	18	Cohort study
Agnew, Hammer et al. [11]	To investigate pressure pain threshold and conditioned pain modulation in ultramarathon runners	Wild Sebastian 100, Saint Sebastian 100 and Keys 100 ultramarathons, Florida, USA; 161 km of sandy trails, concrete sidewalks and gravel paths; Average finishing time of 26 h	23	Cohort study
Schwellnus, Allie et al. [19]	To identify risk factors associated with the development of exercise-associated muscle cramping in ultramarathon runners	Two Oceans ultramarathon, South Africa; 56 km of roads; Cut-off time of 7 h	49	Cohort study
Hoffman, Lee et al. [16]	To investigate (1) pain perception during an ultramarathon race as measured by a pressure pain test and (2) analgesic effect induced by ultramarathon running activity	Western States Endurance Run, California, USA; 161 km of mountain trails; 5500 m of ascent; Cut-off time of 30 h	30	Cohort study
Alschuler, Krabak et al. [9]	To examine the type of pain coping strategies used during ultramarathon races and their effects on performance	Five stages of the 2016 RacingThePlanet 250 km six-stage ultramarathon races in the Atacama Desert in Chile, Gobi Desert in China and Namibian Desert; 40 km (first four stages) and 80 km (fifth stage)	204	Cross-sectional study
Roebuck, Urquhart et al. [18]	To examine pain-related psychological processes in ultramarathon runners; To explore the association between psychological factors and the elevated pain tolerance of these runners	not race-specific	40	Cross-sectional study
Degache, Morin et al. [22]	To examine changes of running mechanics during an ultramarathon	Tor des Géants, France and Italy; 330 km of mountain trails; 24,000 m of ascent; Cut-off time of 150 h	24	Cross-sectional study
Freund, Weber et al. [17]	To compare pain tolerance and personality traits between ultramarathon athletes and a control group	not race-specific	22	Cross-sectional study
Cole, Bontemps et al. [29]	To investigate motivation, prevalence and perceived risks and benefits of NSAID use in ultramarathon runners	not race-specific	167	Case-series study
dos Santos Bunn, de Souza Terra et al. [30]	To analyse the effects of three different physical therapy procedures (massage, stretching, cold-water immersion) on acute pain in ultramarathon runners	2013 to 2015 editions of the Ultramarathon Rio 24 h, Brazil; 400 m loop running track	512	Case-series study
Alschuler, Kratz et al. [38]	To examine reported pain intensity and pain coping strategies during an ultramarathon; To investigate how stage-to-stage changes in pain coping strategies relate to both the amount of time spent thinking about pain and the amount of pain-related interference	Five stages of the 2016 RacingThePlanet 250 km six-stage ultramarathon races in the Atacama Desert in Chile, Gobi Desert in China and Namibian Desert; 40 km (first four stages) and 80 km (fifth stage)	204	Case-series study
Degache, Serain et al. [25]	To investigate the change in postural control during a mountain ultramarathon	Tor des Géants, France and Italy; 330 km of mountain trails (race interrupted at km 206 due to bad weather); 24,000 m of ascent; Cut-off time of 150 h	16	Case-series study
Rochat, Hauw et al. [36]	To find patterns in discussion topics of an online community forum; To understand how discussions improve training and races	not race-specific	155	Case-series study
Didier, Vauthier et al. [26]	To explore the use of medication among participants in an ultramarathon	Infernal Trail des Vosges, France; 72 km (race A) and 160 km (race B) of mountain trails; 3000 m (race A) and 7300 m (race B) of ascent	297	Case-series study
Martínez, Aguiló et al. [27]	To evaluate and compare consumption of non-steroidal anti-inflammatory drugs before, during and immediately after three mountain ultramarathon runs of various distances	Ultra Mallorca Serra de Tramuntana, Spain; 44 km (Marathon), 67 km (Trail) and 112 km (Ultra) of mountain trails; 1424 m (Marathon), 2521 m (Trail) and 4450 m (Ultra); Average finishing time of 6.2 h (Marathon), 10.4 h (Trail) and 17.5 h (Ultra)	238	Case-series study
Visconti, Capra et al. [31]	To investigate the effects of massage on pain in runners suffering from delayed-onset muscle damage during an ultramarathon	Tor des Géants, France and Italy; 330 km of mountain trails; 24,000 m of ascent; Cut-off time of 150 h	25	Case-series study
Schütz, Schmidt-Trucksäss et al. [24]	To demonstrate the feasibility of conducting a field study based on mobile magnetic resonance imaging measurements in ultramarathon runners; To record adaptive responses of tissues, organs and functional systems in these runners	TransEurope FootRace, in multiple countries in Europe; 4487 km of roads; Fixed duration of 64 days	44	Case-series study
Hoffman and Fogard [5]	To explore demographic characteristics and issues that affect performance in an ultramarathon race	Western States Endurance Run (WSER) and Vermont Endurance Race (VER), USA; 161 km of trails; 5500 m (WSER) and 4300 m (VER) of ascent; Cut-off time of 30 h	500	Case-series study
Morin, Tomazin et al. [23]	To investigate changes in running mechanics in a mountain ultramarathon race	Ultra-Trail du Mont-Blanc, France and Italy; 166 km of mountain trails; 9500 m of ascent; Average finishing time of 38 h	18	Case-series study
Gajda, Samełko et al. [37]	To identify the factors enabling a runner to win a 24 h ultramarathon race	24 h ultramarathon; Poland; 2-km loop running track	1	Single-case report
Sadowska-Krępa, Kłapcińska et al. [32]	To present a case of chest pain leading to withdrawal	48 h ultramarathon race in Poland; 129 km of completed distance on a 3 km loop running track	1	Single-case report
Pasternak, Ellero et al. [34]	To present a case of effort rupture of oesophageal wall during an ultramarathon	Western States Endurance Run, California, USA; 161 km of mountain trails; 5500 m of ascent; Cut-off time of 30 h	1	Single-case report
Best, Barwick et al. [35]	To compare physiological status, psychological condition and nutritional intake of the same runner during two 100-mile running races	161 km race; first trial with withdrawal and 2 years later second trial with completion	1	Single-case report
Cope and Kropelnicki [33]	To present a case of ocular freezing and abrasion during an ultramarathon	7-day non-stop ultramarathon, UK; Withdrawal at km 67 on day 1	1	Single-case report
Bennett, Ratzlaff et al. [4]	To explore how seven women ultrarunners narrate their experiences of pain	Privately funded sponsored event; 4.1 km loop around a lake; Cut-off time of 6 days	7	Qualitative study
Rochat, Gesbert et al. [7]	To identify phenomenological characteristics shared by ultramarathon runners; To explore meaningful activities of runners during the race	Tor des Géants, France and Italy; 330 km of mountain trails; 24,000 m of ascent; Cut-off time of 150 h	10	Qualitative study
Antonini, Rochat et al. [6]	To report on the experience of race withdrawal by ten ultramarathon runners	Grand Raid de la Réunion; 65 km (short race), 97 km (middle race) and 173 km (long race) of nature trails; 3922 m (short), 5655 m (middle) and 9996 m (long) of ascent	10	Qualitative study
Holt, Lee et al. [20]	To examine the individual experiences of participants in an ultramarathon	Canadian Death Race; 125 km of mountain trails; 5100 m of ascent; Cut-off time of 24 h	6	Qualitative study
Hanold [21]	To understand how (1) the physical body of female ultramarathon runners becomes a desired body beyond the race and (2) this desired body produces multiple and complex subjectivities	not race-specific	8	Qualitative study
